# Cyanoacrylate Glue Insertion Versus Seton Placement in the Management of Trans-sphincteric Anal Fistula: A Prospective Comparative Study

**DOI:** 10.7759/cureus.109658

**Published:** 2026-05-25

**Authors:** Sarana Venkata Anuja, Anand Kannan, Sivaraja P.K., Arulappan Thangasamy

**Affiliations:** 1 Department of General Surgery, Sri Ramachandra Institute of Higher Education and Research, Chennai, IND; 2 Department of General Surgery, Sri Ramachandra Medical College and Research Institute, Chennai, IND

**Keywords:** anal fistula, cyanoacrylate glue, seton, sphincter-preserving surgery, trans-sphincteric fistula

## Abstract

Background: Anal fistula is a common perianal condition associated with significant morbidity, recurrence, and risk of incontinence. Traditional surgical techniques such as seton placement are effective but are often associated with pain, prolonged healing time, and extended hospitalisation. Cyanoacrylate glue has emerged as a minimally invasive sphincter-preserving alternative.

Methods: This prospective comparative study was conducted at Sri Ramachandra Institute of Higher Education and Research, Chennai, India, between June 1, 2023, and December 31, 2024. Fifty patients with uncomplicated trans-sphincteric fistula (St James University Hospital classification grade 3) were randomly allocated to receive either seton placement (n=25) or cyanoacrylate glue insertion (n=25). Primary outcomes included postoperative pain measured by visual analogue scale (VAS), duration of hospital stay, healing time, healing rate, and anal incontinence assessed by the Wexner continence score.

Results: The mean age was 45.3 years (SD 8.2) in the seton group and 39.1 years (SD 7.6) in the glue group. Both groups were comparable in demographic characteristics. VAS pain scores were significantly lower in the glue group compared with the seton group (mean 2.0 (SD 0.8) vs 3.4 (SD 1.1); p<0.001). Hospital stay was shorter in the glue group (2.6 days (SD 1.2) vs 5.3 days (SD 1.8); p<0.001). Healing time was significantly reduced with cyanoacrylate glue (2.75 weeks (SD 1.1) vs 5.79 weeks (SD 2.3); p<0.001). Healing rates were 96% (24 of 25) in the seton group versus 80% (20 of 25) in the glue group (p=0.189). No anal incontinence was observed in either group at three-month follow-up.

Conclusion: Cyanoacrylate glue insertion offers a safe, minimally invasive alternative to seton placement for carefully selected patients with uncomplicated trans-sphincteric anal fistula, with significant advantages in pain reduction, shorter hospital stay, and faster healing. Although healing rates were lower with glue (80% vs 96%), this difference was not statistically significant, and the study was underpowered to detect this difference in healing rate as a secondary endpoint. All treatment failures were successfully managed with salvage fistulotomy or repeat glue application without morbidity. Larger randomised controlled trials adequately powered for healing rate as a co-primary endpoint are warranted to confirm long-term efficacy.

## Introduction

Fistula-in-ano represents a chronic abnormal communication between the anorectal canal and perianal skin, most commonly arising from cryptoglandular infection [1]. The condition affects approximately 1.8-2.8 per 10,000 individuals globally, with male predominance and peak incidence in the fourth and fifth decades of life [2]. Despite its benign nature, anal fistula accounts for significant patient morbidity, with recurrence rates ranging from 5% to 30% and substantial risk of anal incontinence following surgical intervention [3]. The primary goals of anal fistula treatment include complete eradication of sepsis, excision of the fistulous tract, prevention of recurrence, and preservation of anal sphincter function [4]. Traditional surgical approaches such as fistulotomy and fistulectomy are effective for simple, low-lying fistulas but carry unacceptable risks of sphincter injury and subsequent incontinence when applied to trans-sphincteric or high fistulas [5]. Seton placement, a technique described as early as the time of Hippocrates, remains the most widely employed surgical method for complex and trans-sphincteric fistulas [6]. Recent studies have demonstrated healing rates ranging from 88% to 100% with seton techniques, particularly when employing loose or cutting setons followed by fistulotomy [7,8]. However, seton placement is associated with several drawbacks, including prolonged healing time (typically four to eight weeks), requirement for multiple tightening sessions, substantial postoperative pain, and the necessity for extended hospital stay [9]. To overcome these limitations, various minimally invasive sphincter-preserving techniques have been developed over the past two decades, including fibrin glue, bioprosthetic plugs, video-assisted techniques, ligation of intersphincteric fistula tract (LIFT), and cyanoacrylate tissue adhesive [10,11]. Among these alternatives, cyanoacrylate glue-particularly n-butyl-2-cyanoacrylate-has garnered increasing attention due to its rapid polymerisation upon contact with tissue fluids, excellent tissue adhesion properties, bacteriostatic effects, and ability to seal the fistulous tract while promoting natural healing [12]. Several studies have reported promising outcomes with cyanoacrylate glue application in anal fistula management. Barillari and colleagues reported a healing rate of 71.4% with Glubran 2 (n-butyl-2-cyanoacrylate and methacryloxysulfolane) in 21 patients with cryptoglandular anal fistulas, with cumulative healing reaching 90.2% after repeat applications [13]. More recent investigations have demonstrated healing rates between 73% and 85% with cyanoacrylate glue, accompanied by significantly reduced pain, shorter hospital stays, and minimal impact on anal continence [14,15]. Despite these encouraging results, direct comparative data between cyanoacrylate glue and traditional seton placement specifically for uncomplicated trans-sphincteric fistulas remain limited. The present study was therefore designed to conduct a prospective head-to-head comparison of these two techniques, evaluating not only efficacy and safety outcomes but also patient-centred endpoints such as postoperative pain and quality of recovery.

## Materials and methods

Study design and participants

This prospective, randomised comparative study was conducted at the Department of General Surgery, Sri Ramachandra Institute of Higher Education and Research, Chennai, India, between June 1, 2023, and December 31, 2024. The study protocol was approved by the Institutional Ethics Committee and conducted in accordance with the Declaration of Helsinki. All participants provided written informed consent before enrollment.

Eligible participants were adults aged 18 years or older with a clinical and radiological diagnosis of uncomplicated trans-sphincteric anal fistula (St James University Hospital classification grade 3), including those with recurrent fistulas after previous surgery. Key exclusion criteria included age younger than 18 years, non-trans-sphincteric fistulas (intersphincteric, suprasphincteric, or extrasphincteric), multiple or complex fistulous tracts, specific aetiologies such as tuberculosis, Crohn's disease, or malignancy, presence of active perianal sepsis, and patient refusal of consent.

Randomisation and blinding

Sample size calculation was performed using earlier comparative studies, determining that a minimum of 22 patients per group would provide 80% power to detect a clinically significant difference in primary outcomes at a two-sided α of 0.05. Accounting for potential attrition, 25 patients were allocated to each group. The study was accordingly powered for its primary endpoints; however, post-hoc calculation confirms that approximately 100 patients per group would be required to detect the observed 16-percentage-point difference in healing rate with 80% power, and future trials should plan for this accordingly.

Procedures

All patients underwent preoperative evaluation, including detailed history, digital rectal examination, proctoscopy, and magnetic resonance imaging of the pelvis to delineate the fistula tract. Bowel preparation was performed with polyethylene glycol solution administered 12-18 hours before surgery. All procedures were performed under spinal anaesthesia with patients in the lithotomy position.

In both groups, the fistula tract was initially identified by injecting methylene blue dye through the external opening and confirmed with a malleable fistula probe passed from the external to the internal opening. The tract was then gently curetted and irrigated with normal saline and diluted hydrogen peroxide (1:1) to remove granulation tissue and debris.

Seton Group

A cutting seton was fashioned using a silastic vessel loop (Penrose drain, 0.25 inch diameter; Medline Industries, Northfield, Illinois, USA), threaded through the fistula tract and secured with multiple knots around the sphincter complex. The seton was tightened to produce firm pressure without excessive tension. Patients were reviewed at weekly outpatient intervals, and the seton was progressively tightened at each visit, producing gradual trans-sphincteric cutting over a period of four to eight weeks. Once the seton cut through and separated spontaneously, the resulting wound was allowed to heal by secondary intention with regular saline irrigation and gauze dressings. Fistula tract tissue progressively divided during the seton-cutting process was submitted for histopathological examination in all seton group patients.

Glue Group

Following curettage and irrigation, the fistula tract was carefully dried with gauze swabs. N-butyl-2-cyanoacrylate tissue adhesive (Histoacryl; B. Braun SE, Melsungen, Germany) was drawn into a 2 mL syringe. A 16-gauge venflon catheter was introduced through the external opening to the level of the internal opening, and the glue was slowly injected while gradually withdrawing the catheter, ensuring complete filling from the internal opening outwards. Gentle pressure was applied for 60 seconds to facilitate polymerisation. The external opening was left open for drainage.

No tissue excision was performed in the glue group. Histopathological material in this group was obtained from curettage specimens (granulation tissue removed during tract preparation) available in 23 of 25 patients; in two patients, insufficient curettage material precluded histopathological analysis.

Although fistulotomy was technically feasible in all enrolled patients (all had uncomplicated grade 3 trans-sphincteric fistulas with adequate sphincter length for safe division), participants were enrolled specifically to evaluate cyanoacrylate glue as a sphincter-preserving first-line alternative to immediate surgery. Fistulotomy was pre-designated as a rescue procedure for treatment failures and was not offered as the index intervention, ensuring that inclusion in the glue arm reflected genuine clinical equipoise between the two study treatments.

Outcomes

The primary outcomes were postoperative pain severity measured using a 10-point visual analogue scale (VAS; 0=no pain, 10=worst imaginable pain) on postoperative days 0, 1, and 2; duration of hospital stay in days; time to complete healing in weeks, defined as complete epithelialisation of the fistula tract with no discharge; and healing rate at three months, defined as the proportion of patients achieving complete closure of both internal and external openings with no evidence of persistent or recurrent fistula on clinical examination and imaging. Secondary outcomes included anal incontinence assessed using the Wexner continence score [16] (range 0-20, with 0 indicating perfect continence) at three months, and histopathological findings from excised fistula tract specimens.

Patients were followed up in the outpatient clinic at two weeks, one month, two months, and three months post-procedure. At each visit, clinical assessment included inspection of the perianal area, digital rectal examination, and evaluation for signs of recurrence or complications.

Statistical analysis

Statistical analyses were performed using IBM SPSS Statistics for Windows, Version 26 (Released 2018; IBM Corp., Armonk, New York, United States). Continuous variables were assessed for normality using the Shapiro-Wilk test and presented as mean±SD for normally distributed data or median (IQR) for non-normally distributed data. Categorical variables were expressed as frequencies and percentages. Between-group comparisons for continuous variables were conducted using an independent samples t-test for normally distributed data or a Mann-Whitney U test for non-normally distributed data. Categorical variables were compared using the χ^2^ test or Fisher's exact test as appropriate. A two-sided p-value of less than 0.05 was considered statistically significant. All analyses were performed on an intention-to-treat basis.

## Results

Between June 1, 2023, and December 31, 2024, 68 patients with trans-sphincteric anal fistula were assessed for eligibility. Of these, 18 were excluded (12 declined participation, four had complex fistulas with multiple tracts, and two had underlying Crohn's disease). The remaining 50 patients were randomised to receive either seton placement (n=25) or cyanoacrylate glue insertion (n=25). All patients completed the three-month follow-up period with no loss to follow-up. The baseline demographic and clinical characteristics of the study participants are presented in Table 1. The mean age was 45.3 years (SD 8.2) in the seton group and 39.1 years (SD 7.6) in the glue group (p=0.112). Male patients constituted 70% of the overall cohort. Both groups were well balanced in terms of age, sex, body mass index, presence of diabetes mellitus, smoking status, and previous fistula surgery history (Table 1).

**Table 1:** Baseline demographic and clinical characteristics Data are presented as mean±SD, n (%), or median (IQR). Test statistics: Age (t=1.615), BMI (t=0.951), distance from anal verge (t=0.662), sex (χ^2^=0.107), diabetes (χ^2^=0.166), smoking (χ^2^=0.114), previous surgery (χ^2^=0.136), duration of symptoms (U=278.5), and fistula location (χ^2^=0.395). IQR: interquartile range; SD: standard deviation

Characteristic	Seton group (n=25)	Glue group (n=25)	p value
Age (years), mean±SD	45.3±8.2	39.1±7.6	0.112
Male sex, n (%)	18 (72)	17 (68)	0.744
Body mass index (kg/m^2^), mean±SD	24.6±3.2	23.8±2.9	0.348
Diabetes mellitus, n (%)	4 (16)	3 (12)	0.683
Smoking, n (%)	7 (28)	6 (24)	0.736
Previous fistula surgery, n (%)	5 (20)	4 (16)	0.715
Duration of symptoms (months), median (IQR)	8 (4-14)	7 (3-12)	0.524
Fistula location, n (%)			
Posterior	16 (64)	17 (68)	0.821
Anterior	5 (20)	4 (16)	
Lateral	4 (16)	4 (16)	
Distance from anal verge (cm), mean±SD	2.8±0.6	2.7±0.5	0.512

Postoperative pain scores, assessed using the VAS, were significantly lower in the glue group compared with the seton group across all time points (Table 2). On postoperative day 0, the mean VAS score was 2.0 (SD 0.8) in the glue group versus 3.4 (SD 1.1) in the seton group (p<0.001). This difference persisted on day 1 (1.8 (SD 0.7) vs 3.2 (SD 1.0); p<0.001) and day 2 (1.2 (SD 0.6) vs 2.8 (SD 0.9); p<0.001). The duration of hospital stay was substantially shorter in the glue group compared with the seton group (mean 2.6 days (SD 1.2) vs 5.3 days (SD 1.8); p<0.001). The majority of patients in the glue group (18 of 25; 72%) were discharged within 48 hours of surgery, whereas only 4 of 25 (16%) in the seton group achieved early discharge (p<0.001). Complete healing, defined as epithelialisation of the fistula tract with no discharge from either opening, occurred significantly earlier in the glue group (mean 2.75 weeks (SD 1.1)) compared with the seton group (mean 5.79 weeks (SD 2.3); p<0.001; Table 2). At three-month follow-up, the healing rate was 96% (24 of 25) in the seton group versus 80% (20 of 25) in the glue group. This difference was not statistically significant (p=0.189). In the seton group, one patient developed persistent discharge requiring surgical revision at eight weeks. In the glue group, five patients experienced treatment failure, manifesting as persistent or recurrent discharge from the fistula tract; four of these patients subsequently underwent successful fistulotomy, while one underwent repeat glue application with successful healing.

**Table 2:** Clinical outcomes and complications Data are presented as mean±SD, n (%), or median (IQR). VAS: visual analogue scale (0-10, higher scores indicate worse pain). Test statistics: VAS day 0 (t=5.14), VAS day 1 (t=5.74), VAS day 2 (t=7.42), hospital stay (t=6.25), healing time (t=5.96), healing rate (Fisher's exact, p=0.189), Wexner score (U=312.5), bleeding (Fisher's exact, p=0.235), infection (χ^2^=1.333), and reoperation (Fisher's exact, p=0.347). IQR: interquartile range; SD: standard deviation

Outcome	Seton group (n=25)	Glue group (n=25)	p-value
Primary outcomes			
VAS pain score, mean±SD			
Postoperative day 0	3.4±1.1	2.0±0.8	<0.001
Postoperative day 1	3.2±1.0	1.8±0.7	<0.001
Postoperative day 2	2.8±0.9	1.2±0.6	<0.001
Hospital stay (days), mean±SD	5.3±1.8	2.6±1.2	<0.001
Healing time (weeks), mean±SD	5.79±2.3	2.75±1.1	<0.001
Complete healing at three months, n (%)	24 (96)	20 (80)	0.189
Secondary outcomes			
Wexner continence score at three months, median (IQR)	0 (0-0)	0 (0-0)	1.000
Complications, n (%)			
Bleeding	3 (12)	0 (0)	0.235
Infection/purulent discharge	2 (8)	6 (24)	0.249
Need for reoperation	1 (4)	4 (16)	0.347

Assessment of anal continence using the Wexner score at three-month follow-up revealed no significant deterioration in either group. All patients in both groups maintained perfect continence (Wexner score 0) at the final assessment. No episodes of flatus incontinence, liquid stool incontinence, or solid stool incontinence were reported in either group. Postoperative complications were infrequent in both groups. In the seton group, three patients (12%) developed minor bleeding from the seton site requiring no intervention beyond reassurance. Two patients (8%) developed wound infection, managed successfully with oral antibiotics. In the glue group, six patients (24%) experienced purulent discharge in the early postoperative period (within two weeks), which resolved with intravenous antibiotic therapy (ceftriaxone 1 g twice daily for five days). No patient in either group required reoperation for complications during the initial three-month follow-up period. Histopathological examination of excised fistula tract specimens (available for all patients in the seton group and 23 of 25 patients in the glue group) revealed nonspecific chronic inflammation in all cases. No evidence of granulomatous disease, malignancy, or specific infections was identified in any specimen.

## Discussion

This prospective comparative study demonstrates that cyanoacrylate glue insertion is associated with significantly reduced postoperative pain, shorter hospital stay, and faster healing compared with traditional seton placement in patients with uncomplicated trans-sphincteric anal fistula. Although the healing rate at three months was numerically lower with glue (80% vs 96%), this difference did not reach statistical significance. Importantly, neither technique was associated with anal incontinence during the follow-up period.

Our findings align with and extend previous reports on cyanoacrylate glue application in anal fistula management. Barillari and colleagues reported a primary healing rate of 71.4% with n-butyl-2-cyanoacrylate in cryptoglandular fistulas, improving to 90.2% after repeat applications [13]. More recently, Sharma and colleagues documented a 73% efficacy rate with cyanoacrylate glue in a cohort of 30 patients with anal fistula, with significant improvement in discomfort scores [15]. Our 80% healing rate falls within this range and supports the viability of cyanoacrylate glue as a treatment option.

The significantly lower pain scores observed with glue insertion merit particular attention. This finding likely reflects the minimally invasive nature of the glue technique, which avoids the mechanical pressure and inflammatory response associated with seton placement. Reduced postoperative pain translates directly to improved patient comfort and satisfaction, reduced analgesic requirements, and earlier return to normal activities. This patient-centred benefit assumes particular importance given the chronicity and psychosocial impact of perianal disease [17].

The markedly shorter hospital stay with glue (2.6 days vs 5.3 days) represents both a clinical and economic advantage. In low-resource settings and healthcare systems with constrained bed availability, techniques that facilitate early discharge without compromising outcomes are especially valuable. The reduced hospitalisation also minimises exposure to healthcare-associated infections and reduces overall treatment costs.

Seton placement demonstrated a higher healing rate (96%) in our series, consistent with contemporary reports. Recent studies from Turkey and Sudan have documented healing rates ranging from 88% to 100% with loose or cutting seton techniques for trans-sphincteric fistulas [7,8,18]. Bük and colleagues reported 100% healing in 114 patients with trans-sphincteric fistula treated with loose seton followed by fistulotomy, with minimal incontinence (1.8%) and low recurrence (4.8%) during a median 27.8-week follow-up [7]. However, these superior healing rates come at the cost of prolonged healing time (median 4-8 weeks), multiple clinic visits for seton tightening, and substantial patient discomfort-drawbacks confirmed by our study.

The absence of anal incontinence in both groups is reassuring and reflects the sphincter-preserving nature of both techniques. Incontinence remains the most feared complication of anal fistula surgery, with rates of 10-24% reported after traditional fistulotomy [19]. Modern sphincter-preserving approaches, including seton drainage and biological glues, have been developed specifically to mitigate this risk. Recent large-scale studies of hybrid and loose seton techniques have documented incontinence rates below 5%, consistent with our zero-incontinence finding [20-22].

Several limitations merit acknowledgement. First, although the sample size was calculated and adequate to detect clinically significant differences in primary outcomes (postoperative pain and hospital stay), the study was underpowered to detect the observed 16-percentage-point difference in healing rate (80% vs 96%). Post-hoc calculation indicates that approximately 100 patients per group would be required to demonstrate this difference with 80% statistical power at α=0.05. The sample size limitation therefore applies specifically to the healing rate comparison, not to the primary outcomes for which the study was prospectively powered. Future trials should incorporate healing rate as a co-primary endpoint and be sized accordingly.

Second, the single-centre design may limit generalisability. Third, the three-month follow-up precludes assessment of long-term recurrence. Fourth, complex fistulas and specific aetiologies were excluded; findings apply specifically to uncomplicated trans-sphincteric fistulas. Fifth, quality-of-life assessment beyond pain scores was not performed. Finally, a cost-effectiveness analysis was not conducted.

Clinical decision-making should balance these trade-offs based on individual patient factors. Cyanoacrylate glue may be particularly beneficial for patients prioritising rapid recovery, minimal pain, and shorter hospitalisation, or those unable to attend multiple clinic visits for seton tightening. Conversely, seton placement may be preferred when maximal healing rate is paramount or when repeat procedures are acceptable to the patient. The repeatability of glue application offers reassurance; in our series, four of five failures were successfully managed with subsequent fistulotomy, while repeat glue injection succeeded in one patient.

Future research priorities include adequately powered randomised controlled trials with sample sizes sufficient to detect clinically meaningful differences in healing rates, extended follow-up periods (minimum 12 months) to assess long-term recurrence, inclusion of complex fistulas and special populations (e.g., Crohn's disease, previous irradiation), cost-effectiveness analyses incorporating direct medical costs and indirect costs (lost productivity, caregiving), quality-of-life assessments using validated instruments, and comparative studies of different tissue adhesive formulations.

## Conclusions

Cyanoacrylate glue insertion represents a safe, effective, minimally invasive alternative to seton placement for carefully selected patients with uncomplicated trans-sphincteric anal fistula. It provides clinically significant advantages in postoperative pain reduction, hospital stay duration, and healing time without compromising anal continence. Although healing rates are lower than with seton placement (80% vs 96%), this difference was not statistically significant, and the study was underpowered for this secondary endpoint. All treatment failures were successfully managed with salvage fistulotomy or repeat glue application without complication, confirming that a glue-first strategy does not compromise subsequent surgical options. For patients seeking rapid recovery with minimal discomfort, cyanoacrylate glue insertion merits consideration as a first-line sphincter-preserving approach for trans-sphincteric fistula-in-ano. Larger, adequately powered multicentre randomised controlled trials with extended follow-up are required to establish long-term efficacy and confirm the healing rate profile.

## References

[1] Parks AG, Gordon PH, Hardcastle JD (1976). A classification of fistula-in-ano. Br J Surg.

[2] Sainio P (1984). Fistula-in-ano in a defined population. Incidence and epidemiological aspects. Ann Chir Gynaecol.

[3] Mei Z, Wang Q, Zhang Y (2019). Risk factors for recurrence after anal fistula surgery: a meta-analysis. Int J Surg.

[4] Williams G, Williams A, Tozer P, Phillips R, Ahmad A, Jayne D, Maxwell-Armstrong C (2018). The treatment of anal fistula: second ACPGBI Position Statement - 2018. Colorectal Dis.

[5] Ritchie RD, Sackier JM, Hodde JP (2009). Incontinence rates after cutting seton treatment for anal fistula. Colorectal Dis.

[6] Seow-Choen F, Nicholls RJ (1992). Anal fistula. Br J Surg.

[7] Bük ÖF, Ocak S, Avcı MA, Akgün C, Bidil MG (2024). Outcomes of loose seton followed by fistulotomy in transsphincteric perianal fistulas: a retrospective study. Turk J Colorectal Dis.

[8] Durgun C, Tüzün A (2023). The use of a loose seton as a definitive surgical treatment for anorectal abscesses and complex anal fistulas. Adv Clin Exp Med.

[9] Hammond TM, Knowles CH, Porrett T, Lunniss PJ (2006). The Snug Seton: short and medium term results of slow fistulotomy for idiopathic anal fistulae. Colorectal Dis.

[10] Emile SH, Khan SM, Adejumo A, Koroye O (2020). Ligation of intersphincteric fistula tract (LIFT) in treatment of anal fistula: an updated systematic review, meta-analysis, and meta-regression of the predictors of failure. Surgery.

[11] de Groof EJ, Cabral VN, Buskens CJ, Morton DG, Hahnloser D, Bemelman WA (2016). Systematic review of evidence and consensus on perianal fistula: an analysis of national and international guidelines. Colorectal Dis.

[12] Singer AJ, Quinn JV, Hollander JE (2008). The cyanoacrylate topical skin adhesives. Am J Emerg Med.

[13] Barillari P, Basso L, Larcinese A, Gozzo P, Indinnimeo M (2006). Cyanoacrylate glue in the treatment of ano-rectal fistulas. Int J Colorectal Dis.

[14] Koli D, Kumar P, Panda V (2020). Evaluation of the role of cyanoacrylate glue in the management of fistula-in-ano. Int Surg J.

[15] Sharma R, Lonare SB, Nagar S, Badhal S, Anand S (2024). Efficacy and efficiency of cyanoacrylate glue in fistula-in-ano. Cureus.

[16] Jorge JM, Wexner SD (1993). Etiology and management of fecal incontinence. Dis Colon Rectum.

[17] Owen HA, Buchanan GN, Schizas A, Cohen R, Williams AB (2016). Quality of life with anal fistula. Ann R Coll Surg Engl.

[18] Elnaim Ali ALK, Wong MP, Sagap I (2022). The value of cutting seton for high transsphincteric anal fistula in the era of its misery. Malays J Med Sci.

[19] Jordán J, Roig JV, García-Armengol J, García-Granero E, Solana A, Lledó S (2010). Risk factors for recurrence and incontinence after anal fistula surgery. Colorectal Dis.

[20] Ege B, Leventoglu S, Mentes BB, Yilmaz U, Oner AY (2014). Hybrid seton for the treatment of high anal fistulas: results of 128 consecutive patients. Tech Coloproctol.

[21] Chen C, Wang W, Mei X (2024). Closed trans-intersphincteric fistulotomy: a new modified sphincter-sparing technique for high transsphincteric anal fistula. Front Surg.

[22] Emile SH, Elfeki H, Thabet W (2017). Predictive factors for recurrence of high transsphincteric anal fistula after placement of seton. J Surg Res.

